# The effects of sonic hedgehog signaling pathway components on non-small-cell lung cancer progression and clinical outcome

**DOI:** 10.1186/1477-7819-12-268

**Published:** 2014-08-21

**Authors:** Jinwook Hwang, Myoung Hee Kang, Young A Yoo, Yu Hua Quan, Hyun Koo Kim, Sang Cheul Oh, Young Ho Choi

**Affiliations:** Department of Thoracic and Cardiovascular Surgery, Korea University Ansan Hospital, Korea University College of Medicine, 123 Jeokgeum-ro, Danwon-gu, Ansan, Gyeonggi-do, 425-707 Republic of Korea; Division of Oncology/Hematology, Departments of Internal Medicine, Korea University Guro Hospital, Korea University College of Medicine, 97 Guro-dong kil, Guro-ku, Seoul, 152-703 Republic of Korea; Department of Thoracic and Cardiovascular Surgery, Korea University Guro Hospital, Korea University College of Medicine, 97 Guro-dong kil, Guro-ku, Seoul, 152-703 Republic of Korea

**Keywords:** Gli1, Hedgehog pathway, LYVE-1, NSCLC, Prognosis, Sonic hedgehog, VEGF-D

## Abstract

**Background:**

Researchers in recent studies have reported that the sonic hedgehog (Shh) signaling pathway plays a crucial role during tumorigenesis, angiogenesis and cellular differentiation. We investigated the clinical and pathological significances of the Shh pathway and of its lymphangiogenic components in non-small-cell lung cancer (NSCLC), namely, Shh, glioma-associated oncogene homolog zinc finger protein 1 (Gli1), lymphatic vessel endothelial hyaluronan receptor 1 (LYVE-1) and vascular endothelial growth factor D (VEGF-D).

**Methods:**

The expression of Shh, Gli1, LYVE-1 and VEGF-D in primary NSCLC tissue from 40 patients was examined using immunohistochemical assays, and relationships between expression and clinicopathological data, such as age, gender, histology, tumor size, nodal stage, visceral pleural invasion, lymphatic thromboembolism, recurrence and overall survival were investigated.

**Results:**

Of the 40 specimens examined, 25 (62.5%), 20 (50.0%), 11 (27.5%) and 20 (50.0%) were positive for Shh, Gli1, LYVE-1 or VEGF-D expression, respectively. The expression of Gli1 and LYVE-1 were significantly associated (*P* = 0.011), and Shh and LYVE-1 expression was related to visceral pleural invasion and lymphatic thromboembolism, respectively (*P* < 0.05). Shh expression levels compared on survival curves were statistically significant in univariate logrank analysis (*P* = 0.020). However, other clinicopathological factors did not reveal any statistical significance in univariate and multivariate analyses.

**Conclusions:**

To our knowledge, this the first report of the relationship between components of the Shh signaling pathway and prognosis in NSCLC. The expression of Shh, Gli1 and LYVE-1 was found to be associated with clinicopathological factors and survival. Thus, the overexpression of the Shh signaling pathway could serve as a predictor of malignant behavior, including lymphangiogenesis, in NSCLC.

## Background

Lung cancer is one of the most common causes of cancer-related mortality worldwide [1], and, though its incidence is decreasing among men, the disease shows an increasing trend among women [2]. Air pollution and smoking are well-known etiologies of lung cancer. The only curative treatment modality is complete surgical resection, but many patients have advanced disease with distant metastasis at initial presentation. In such patients, the frequency of recurrence is high after complete resection. Accordingly, the prognosis of lung cancer is dismal, with an overall 5-year survival rate of less than 15%. Owing to the minimal improvements achieved over the past 30 years, a new molecular targeting strategy is needed [3, 4].

The early involvement of the lymphatic system in lung cancer is a probable cause of its poor prognosis, and the presence of lymph node metastasis (N station) is a clinically important prognostic factor that influences therapeutic strategies [5, 6]. Lung cancer can spread in three ways: through the lymphatic and vascular systems and by direct invasion. In the lymphatic system, tumor cells metastasize to intrapulmonary, mediastinal or extrathoracic lymph nodes [5]. If intrapulmonary lymph node metastasis (the N1 station) is confirmed, indicating lymphatic metastasis is limited to one lung, the prognosis is better than that for mediastinal lymph node metastases (the N2 station), which indicates tumor cell spread to the axial lymphatic system and possible metastasis to the contralateral lung or extrathoracic organs [6]. The mechanism of early tumor spread and ease of lymphatic involvement has not been elucidated, although lymphangiogenesis has been suggested to be involved in cancer metastasis with angiogenesis [7]. Several genes are involved in lymphangiogenesis, and tumors can provoke lymphatic capillary growth by producing lymphangiogenic factors, such as the lymphatic vessel endothelial hyaluronan receptor 1 (LYVE-1) members of the vascular endothelial growth factor (VEGF) family. Furthermore, it has been shown that the activity of tumor-induced lymphangiogenesis is directly correlated with the extent of tumor spread to regional lymph nodes [8, 9]. In addition, the expression of sonic hedgehog (Shh) and glioma-associated oncogene homolog zinc finger protein 1 (Gli1) has been shown to affect lymphatic metastasis in cancer [10]. In a prior study, the same authors showed that Shh affects the invasion and motility of cancer cells [11].

The Shh signaling pathway has been shown to play a crucial role in the organogenesis of several organs [12] and has been implicated in the regulation of stem-cell fate as well as tissue repair and regeneration [13, 14]. Shh signaling is activated by binding between Shh and its receptor, Patched (Ptch), which relieves the Ptch-mediated repression of smoothened (Smo), a downstream membrane protein related to G protein–coupled receptors. Upon activation, Smo promotes the nuclear translocations of a family of transcription factors (Cubitus interruptus (Ci) in *Drosophila* and Gli1, Gli2 and Gli3 in vertebrates) and subsequently activates target genes through Gli transcription factors [5]. Furthermore, the constitutive activation of the Shh signaling pathway has been reported in various cancers, including basal cell carcinoma prostate, gastrointestinal and lung cancer [15–19].

Researchers in previous studies have suggested that expression of the Shh signaling pathway could affect the prognosis of patients with epithelial cell cancer [20–22]. However, no report has been issued on the relationship between the Shh signaling pathway and prognosis in non-small-cell lung cancer (NSCLC). Accordingly, our objectives in this study were to investigate the relationship between the expression of Shh-associated factors and lymphangiogenic factors and to determine the prognostic roles of these biomarkers in patients with lung cancer.

## Methods

### Patients and tissue specimens

We collected formalin-fixed, paraffin-embedded samples from 40 patients with NSCLC (adenocarcinoma = 15, squamous cell carcinoma = 20, mixed type = 2, bronchioloalveolar carcinoma = 3) who had undergone surgical treatment for NSCLC without neoadjuvant treatment at Korea University Guro Hospital between 2007 and 2009. Permission from the institutional review board of Guro Hospital was obtained beforehand, and informed consent was obtained from all patients (KUGGR-2010-057).

All patients were followed for a median 35.5 months (range = 1 to 54 months). The sample comprised 30 men and 10 were women with an overall mean age of 62.8 years (range = 44 to 82 years). Slides were reviewed by two pathologists, and histological type and tumor grade were confirmed according to the 2004 World Health Organization classification of lung cancer. The pathologic tumor and nodal status according to TNM classification were obtained from primary pathology reports. Sixteen (40.0%) of the forty patients had stage IA cancer, and eleven (27.5%) were in stage IB. Lymph node involvement (above stage IIA) was present in 13 patients (32.5%).

### Immunohistochemistry

Representative formalin-fixed, paraffin-embedded tissue sections (5 μm thick) were used for immunohistochemistry. Tissue sections were first deparaffinized in xylene and then rehydrated using an ethanol series. Antigen retrieval was performed using a microwave oven for 20 minutes in 0.01 M citrate buffer (pH 6.0), followed by treatment with 0.025% trypsin in 50 mM Tris buffer for 5 minutes. Endogenous peroxidase activity was blocked using 3% hydrogen peroxide in phosphate-buffered saline (PBS) for 12 minutes. Sections were then incubated for 1 hour at room temperature in a protein-blocking solution consisting of PBS (pH 7.5) containing 5% normal swine serum and then incubated at 4°C overnight with different primary antibodies. The antibodies, sources, clones and dilutions used are listed in Table 1.

**Table 1:** **Antibodies used for immunohistochemical staining** ^**a**^

Antibody	Source	Clone number	Dilution
Shh	Abcam	EP1190Y	1:200
Gli1	Abcam	Polyclonal antibody	1:50
LYVE-1	Neo-Markers	SPM471	1:30
VEGF-D	Cell Signaling Technology	Polyclonal antibody	1:50

^a^Gli1, Glioma-associated oncogene homolog zinc finger protein 1; LYVE-1, Lymphatic vessel endothelial hyaluronan receptor 1; Shh, Sonic hedgehog; VEGF-D, Vascular endothelial growth factor D.

**Figure 1:**
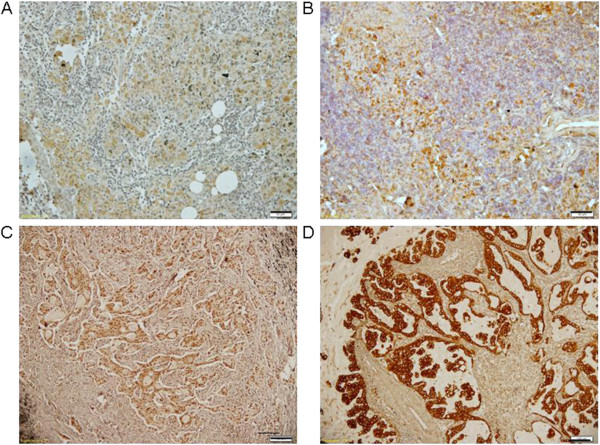
**Activation of the Shh signaling pathway and lymphangiogenesis in non-small-cell lung cancer.** Immunohistochemical staining was performed on tumor tissues obtained from 40 non-small-cell lung cancer (NSCLC) patients for the Sonic hedgehog (Shh) signaling pathway (Shh, glioma-associated oncogene homolog zinc finger protein 1 (Gli1)) and lymphangiogenic factors (vascular endothelial growth factor D (VEGF-D), lymphatic vessel endothelial hyaluronan receptor 1 (LYVE-1)) using appropriate antibodies. Immunohistochemistry scores (IHC scores) were calculated from positive staining area percentages and intensity scores. Staining intensity scores were graded as follows: 0 = negative **(A**), 1 = weak **(B)**, 2 = moderate **(C)** and 3 = strong **(D)**. Scale bar = 50 μm. Positive area percentages were graded as follows: grade 0 (≤5%), 1 (6% to 15%), 2 (16% to 25%), 3 (26% to 50%), 4 (51% to 75%) and 5 (>75%). Positivity was determined using the following formula: IHC score = percentage score × staining intensity score. Positive expression was defined as an IHC score >6.

Next, the sections were washed three times with distilled water, counterstained with Mayer’s hematoxylin (Biogenex Laboratories, San Ramon, CA, USA) and washed with distilled water followed by PBS. Slides were mounted using a universal mounting medium (Dako, Glostrup, Denmark) and examined using a bright-field microscope. Negative controls were prepared in all cases by omitting primary antibodies. The expression levels of Shh, Gli1, LYVE-1 and VEGF-D protein were classified into four groups by staining intensity (intensity scores): 0 (negative), 1 (weak), 2 (moderate) and 3 (strong). The percentages of positive cells were graded (percentage scores) as 0 (<5%), 1 (6% to 15%), 2 (16% to 25%), 3 (26% to 50%), 4 (51 to 75%) or 5 (>75%). Positivity was determined using the following formula: immunohistochemistry score (IHC) = percentage score × intensity score. An IHC score >6 was defined as positive [23] (Figure 1).

### Cell lines and reagents

The human NSCLC cell lines H1299 and Calu-1 were purchased from the Korea Cell Line Bank (KCLB) (Seoul, Korea). Cells were maintained according to instructions provided by the KCLB. Recombinant human Shh N-terminal peptide (N-Shh) was purchased from R&D Systems (Minneapolis, MN, USA), and cyclopamine-KAAD was purchased from Calbiochem (San Diego, CA, USA).

### RT-PCR analysis

Total RNA extraction was performed using TRIzol reagent (Life Technologies, Rockville, MD, USA) according to the manufacturer’s instructions. Transcripts were amplified using 2 μg/μl total RNA by RT-PCR using Moloney murine leukemia virus reverse transcriptase (Gibco/Life Technologies, Gaithersburg, MD, USA) and oligo(dT) 15 primer (Roche Molecular Diagnostics, Indianapolis, IN, USA) (Table 2). β-actin was synthesized by the Bioneer Corporation (Daejeon, Korea). The cycling conditions were 95°C for 10 minutes, followed by 30 amplification cycles of 95°C for 45 seconds, 60°C for 30 seconds and 72°C for 30 seconds. Experiments were repeated three times, and the intensities of DNA bands in agarose gel were quantified using ImageJ software (National Institutes of Health, Bethesda, MD, USA).

**Table 2:** **Primer sequences used for RT-PCR** ^**a**^

	Primers 5′-3′	
**RNA**	**Forward**	**Reverse**
LYVE-1	CCA GTG AGC CGA CAG TTT GGA G	CAG GTA TTG TAG AGT AAG GGG ATG CC
VEGF-D	CAG TGA AGC GAT CAT CTC AG	TAC GAG GTG CTG GTG TTC ATA C
Gli1	TGC CTT GTA CCC TCC TCC CGA A	GCG ATC TGT GAT GGA TGA GAT TCC C
Gli2	AGA TTC TGA GCC AGC AGA GG	TGG TGT CAC TCA GAC AGT TGC
Gli3	TGC AGG GTG AAT GGT ATC AA	TGA TTA GCA CCT GGG GAA AG
β-actin	ACC CAG ATC ATG TTT GAG AC	GGA GTT GAA GGT AGT TTC GT

^a^Gli1, Glioma-associated oncogene homolog zinc finger protein 1; LYVE-1, Lymphatic vessel endothelial hyaluronan receptor 1; Shh, Sonic hedgehog; VEGF-D, Vascular endothelial growth factor D.

### Small interfering RNA transfection

siRNA duplex specific to Gli1, Gli2 and Gli3 was synthesized at Invitrogen (Carlsbad, CA, USA) (Table 3). Scrambled siRNA duplex was used as a nonspecific control siRNA. Transfection was performed using Lipofectamine RNAiMAX reagent (Invitrogen), according to the manufacturer’s instructions.

**Table 3:** **Transfected siRNA sequences** ^**a**^

siRNA	Sequences
Gli1	5′-AUA UCU UGC CCG AAG CAG GUA GUG C-3′
	5′-GCA CUA CCU GCU UCG GGC AAG AUA U-3′
Gli2	5′-CAU AGA UGA CCA CCU CAG CCU CCU G-3′
	5′-CAG GAG GCU GAG GUG GUC AUC UAU G-3′
Gli3	5′-UUC AGU CGC GGA AAC AUU CCA UUC A-3′
	5′-UGA AUG GAA UGU UUC CGC GAC UGA A-3′

^a^Gli1, Glioma-associated oncogene homolog zinc finger protein 1; LYVE-1, Lymphatic vessel endothelial hyaluronan receptor 1; Shh, Sonic hedgehog; VEGF-D, Vascular endothelial growth factor D.

### Cell proliferation assay

Gli1, Gli2 and Gli3 siRNA transfected cells were seeded at a concentration of 4 × 10^3^ cells per 100 μl of culture medium per well in 96-well plates. After 24 or 48 hours, viable cells were counted in triplicate wells using a 3-(4,5-dimethylthiazol-2-yl)-2,5-diphenyltetrazolium bromide (MTT) assay (Roche Molecular Diagnostics) according to the manufacturer’s instructions.

### Statistical analysis

Fisher’s exact test was used to evaluate associations between protein expression and clinical parameters (when two independent groups were compared). The Kaplan-Meier method was used to plot survival curves, and a logrank test was used to compare survival. Multivariate analysis was performed using the Cox proportional hazards model to determine the significance of relationships between variables and survival. SPSS version 15.0 for Windows software (IBM SPSS, Chicago, IL, USA) was used for statistical analysis, and statistical significance was accepted for *P*-values <0.05.

## Results

### Immunohistochemistry of Shh, Gli1, LYVE-1 and VEGF-D in non-small-cell lung cancer

Shh expression was positive in 25 (62.5%) of the 40 NSCLC specimens, and Gli1, LYVE-1 and VEGF-D were positive in 20 (50.0%), 11 (27.5%) and 20 (50.0%) of the 40 specimens, respectively. Normal potions of NSCLC tissues and negative controls were negative for Shh, Gli1, LYVE-1 and VEGF-D (Figure 1).

### Relationships between Shh, Gli 1, LYVE-1 and VEGF-D

The expression of Gli1 and LYVE-1 was correlated (*r* = 0.579, *P* < 0.05) (Table 4). In addition, the expression of Shh, Gli1, LYVE-1 and VEGF-D proteins was found to be related with some clinicopathological factors (Table 5). In particular, Shh was significantly highly expressed in cases of visceral pleural invasion (*P* = 0.01), and Gli was significantly and highly expressed in squamous cell histology (*P* = 0.048). LYVE-1 (the lymphangiogenic factor) was highly expressed in cases of lymphatic thromboembolisms (*P* = 0.001). However, Shh and Gli expression were not found to be related with any strong prognostic factor, such as TNM stage, tumor recurrence or distant metastasis. Furthermore, lymphangiogenic factors LYVE-1 and VEGF-D showed no relation with T stage, N stage, tumor recurrence or distant metastasis.

**Table 4:** **Correlation analysis** ^**a**^

Variable	Shh expression	Gli1 expression	LYVE-1 expression	VEGF-D expression
Shh expression	1			
Gli1 expression	−0.281	1		
LYVE-1 expression	−0.084	0.579*	1	
VEGF-D expression	−0.198	0.169	0.096	1

^a^Gli1, Glioma-associated oncogene homolog zinc finger protein 1; LYVE-1, Lymphatic vessel endothelial hyaluronan receptor 1; Shh, Sonic hedgehog; VEGF-D, Vascular endothelial growth factor D. *P < 0.05.

**Table 5:** **Relationship between the protein expression of Shh, Gli1, LYVE-1 and VEGF-D and non-small-cell lung cancer** ^**a**^

Clinicopathological features	Shh expression			Gli1 expression			LYVE-1 expression			VEGF-D expression		
	Negative	Positive	***P***	Negative	Positive	***P***	Negative	Positive	***P***	Negative	Positive	***P***
Age, yr												
<65	11	12	0.117	11	12	0.749	18	5	0.477	14	9	0.11
≥65	4	13		9	8		11	6		6	11	
Gender												
Female	4	6	1.00	5	5	1	6	4	0.418	5	5	1.00
Male	11	19		15	15		23	7		15	15	
Histology												
Nonsquamous	8	12	0.744	14	6	0.048^†^	15	5	0.723	10	10	1.00
Squamous	7	13		6	14		14	6		10	10	
Tumor size												
<3 cm	11	14	0.273	12	13	0.744	19	6	0.716	13	12	0.744
≥3 cm	4	11		8	7		10	5		7	8	
Node stage												
0	9	18	0.498	12	15	0.311	21	6	0.451	14	13	0.736
1 to 2	6	7		8	5		8	5		6	7	
VPI												
No	13	11	0.01^†^	12	12	1.00	17	7	1.00	12	12	1.00
Yes	2	14		8	8		12	4		8	8	
LTE												
No	13	22	1.00	17	18	1.00	29	6	0.001^†^	18	17	1.00
Yes	2	3		3	2		0	5		2	3	
Smoking history												
No	7	12	0.935	10	9	0.752	13	6	0.583	10	9	0.752
Yes	8	13		10	11		16	5		10	11	
Tumor recurrence												
No	11	18	1.00	13	16	0.288	22	7	0.455	14	15	0.723
Yes	4	7		7	4		7	4		6	5	
Distant metastasis												
No	14	23	1.00	19	18	1.00	27	10	1.00	19	18	1.00
Yes	1	2		1	2		2	1		1	2	

^a^Gli1, Glioma-associated oncogene homolog zinc finger protein 1; LTE, Lymphatic thromboembolism; LYVE-1, Lymphatic vessel endothelial hyaluronan receptor 1; Shh, Sonic hedgehog; VEGF-D, Vascular endothelial growth factor D; VPI, Visceral pleura invasion. P-values were calculated using Fisher’s exact test. ^†^ *P* < 0.05.

### Sonic hedgehog–mediated non-small-cell lung cancer cell line proliferation *in vitro*

**Figure 2:**
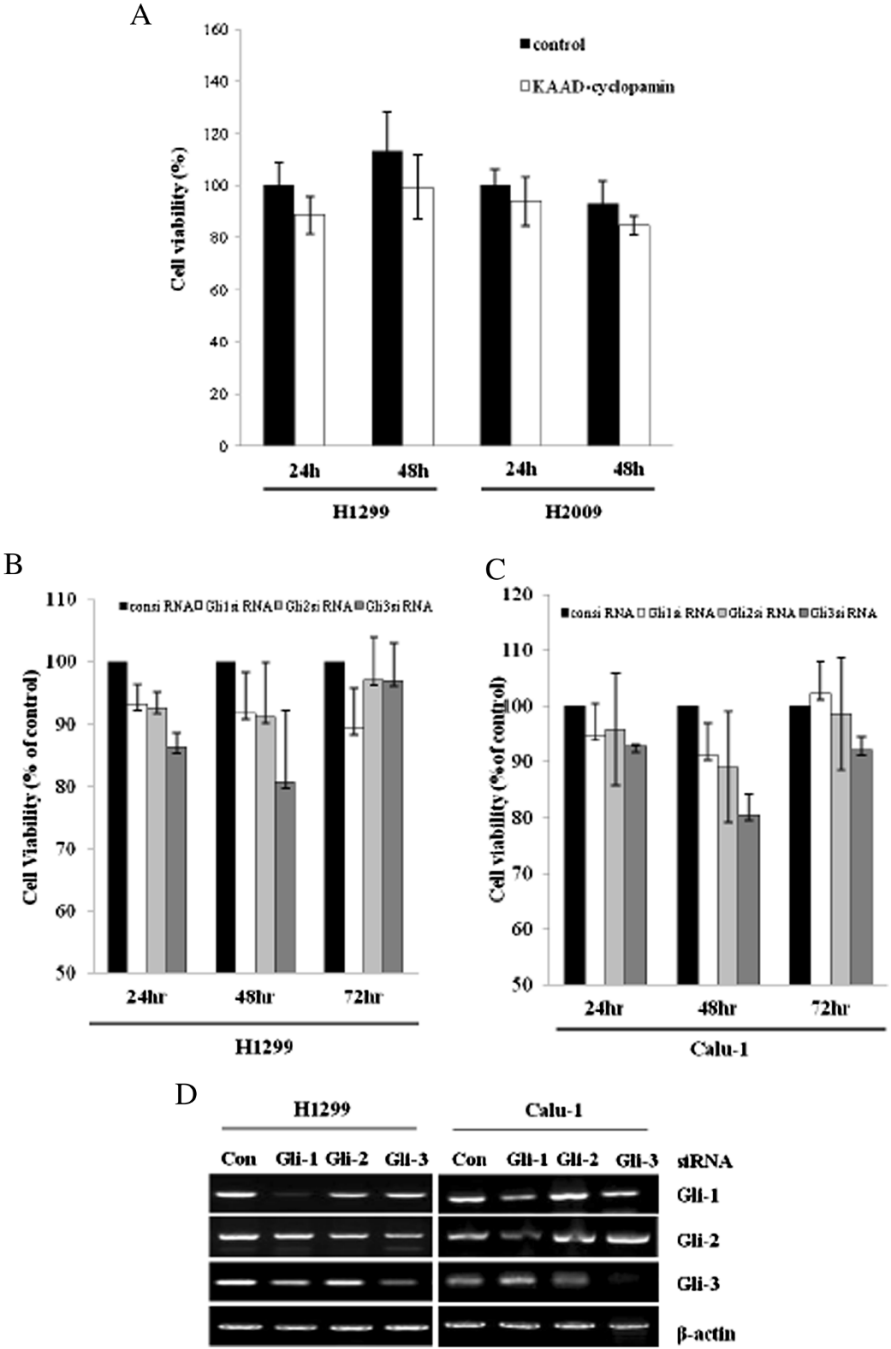
**Sonic hedgehog signaling and non-small-cell lung cancer cell proliferation.** 3-(4,5-dimethylthiazol-2-yl)-2,5-diphenyltetrazolium bromide viability assays were performed to measure cell proliferation. **(A)** Proliferation of H1299 and H2009 cells treated with cyclopamine-KAAD (10 μM). **(B)** and **(C)** Proliferation of H1299 and Calu-1 cells transfected with Gli1, Gli2 or Gli3 siRNA or nonspecific control (Con) siRNA. Bars represent the standard deviations of three independent experiments conducted in triplicate. **(D)** H1299 and Calu-1 cells were transfected with Gli1, Gli2 or Gli3 siRNA or nonspecific control siRNA and harvested 48 hours later. RT-PCR was performed for endogenous Gli1, Gli2 and Gli3 transcripts. β-actin was used as a loading control.

**Figure 3:**
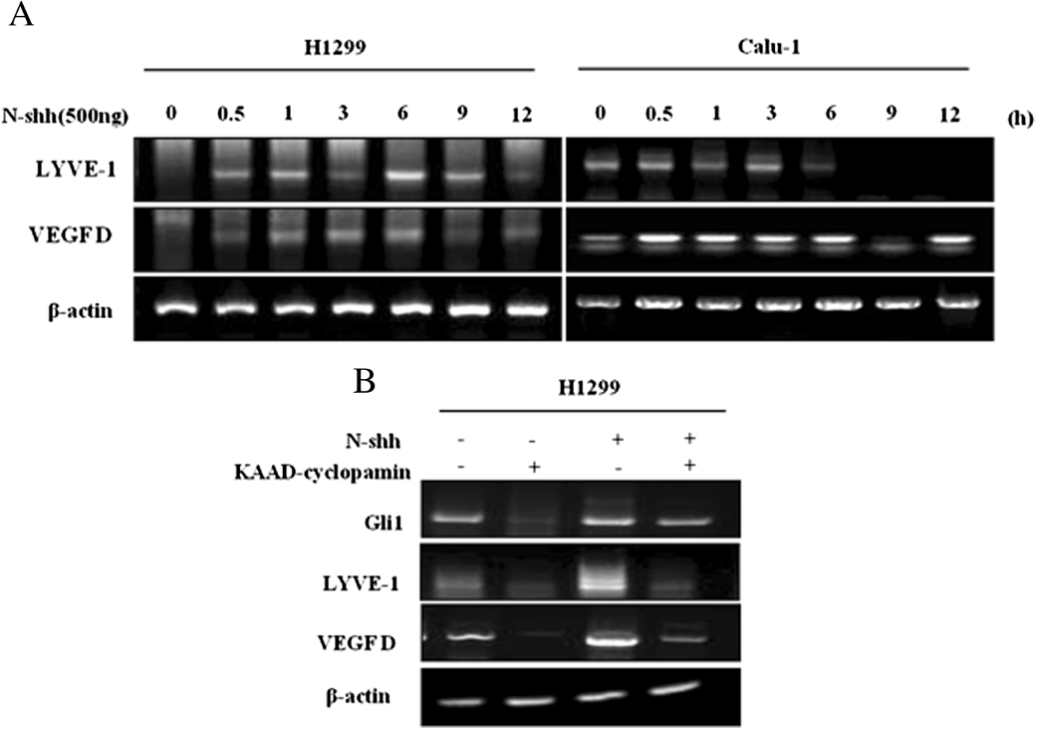
**The sonic hedgehog signaling pathway was regulated by lymphangiogenic factors. (A)** H1299 and Calu-1 cells were treated with 500 ng/ml N-Shh for the indicated times. The total RNA was harvested and subjected to RT-PCR using lymphatic vessel endothelial hyaluronan receptor 1 (LYVE-1), vascular endothelial growth factor D (VEGF-D) or β-actin (loading control) primers. **(B)** H1299 cells were treated with 500 ng of N-Shh or 10 μM cyclopamine-KAAD for 24 hours, and RNAs were isolated and subjected to RT-PCR for Glioma-associated oncogene homolog zinc finger protein 1 (Gli1), LYVE-1, VEGF-D or β-actin (loading control).

To investigate the role of the Shh signaling pathway in the cell proliferation of NSCLC cell lines, we used an MTT assay to examine the effects of Shh on cell proliferation after inhibiting the Shh signaling pathway in H1299 and H2009 cells. As shown in Figure 2A, cyclopamine-KADD (a Smo inhibitor) inhibited the cell growth of both NSCLC cell lines. Gli1, Gli2 and Gli3 siRNA transfectants reduced the proliferation of cells. After 24 and 48 hours, H1299 and Calu-1 cells showed inhibited cell growth versus control siRNA transfected cells (Figures 2 and 3). These observations confirm that inhibition of the Shh signaling pathway regulates the viability of NSCLC cell lines.

### Sonic hedgehog signaling pathway and lymphangiogenesis of non-small-cell lung cancer cell lines *in vitro*

To confirm the contribution made by the Shh signaling pathway to the expression of lymphangiogenic factors in NSCLC, cells were treated with N-Shh peptide or KADD-cyclopamine. Those cells were treated with N-Shh. RT-PCR analysis showed that the mRNA expression of LYVE-1 and VEGF-D in H1299 and Calu-1 cells was induced after 30 minutes of N-Shh stimulation (Figure 3A). However, the blockade of Shh signaling by cyclopamine completely prevented these increases in mRNA expression of LYVE-1 and VEGF-D (Figure 3B). Together, these results show that lymphangiogenic factors are regulated by the Shh signaling pathway.

**Figure 4:**
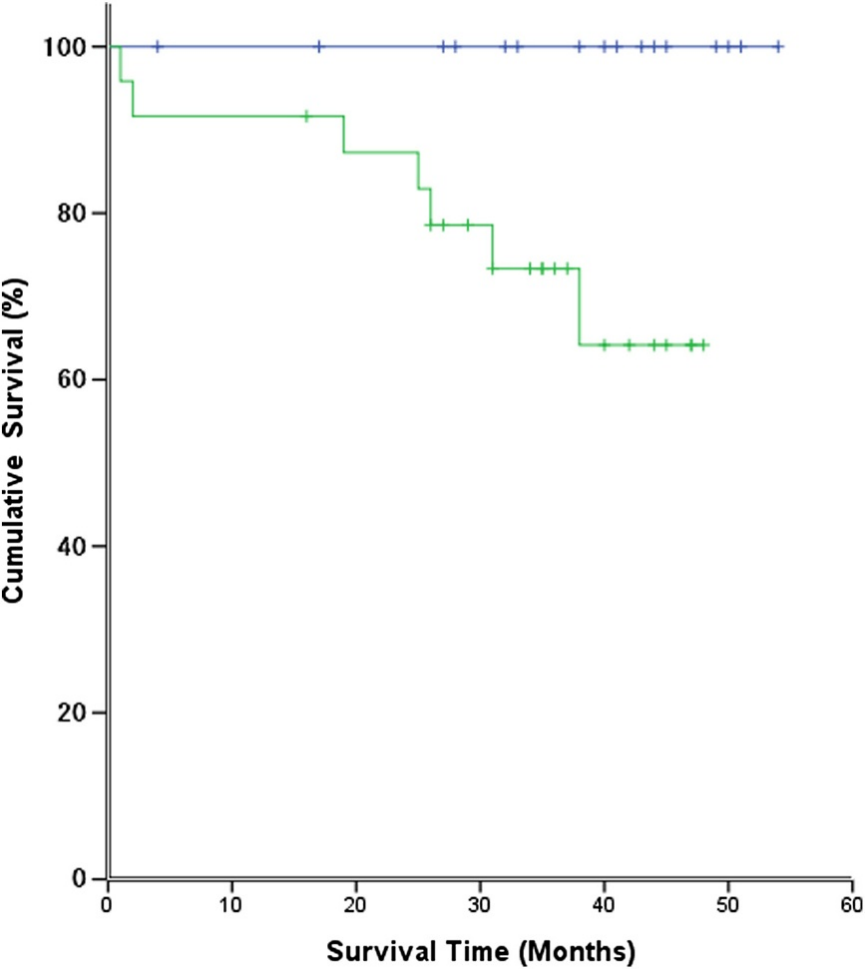
**Overall survival curves for 40 non-small-cell lung cancer patients positive or negative for sonic hedgehog expression.** Survival was analyzed using the Kaplan-Meier method, and survival differences between the positive and negative groups were compared using a logrank test. Patients with a tumor positive for Shh were found to have significantly poorer survival (*P* = 0.020).

### Survival analysis

In the Kaplan-Meier survival analysis, overall 3-year survival was 83.6%. Median survival could not be calculated, because 7 of 40 patients died and 9 were lost to follow-up during the period. We also analyzed relationships between patient survival (all 40 had undergone curative resection) and the expression of Shh, Gli1, LYVE-1 and VEGF-D. Survival curves were plotted for those positive or negative for Shh expression (Figure 4). We found that survival of patients whose Shh expression was positive was significantly lower than patients whose Shh expression was negative. (*P* = 0.020 by logrank analysis). In survival curve analysis of Gli1 expession, there was no statistical significance (*P* = 0.55 by logrank analysis). However, these indicated that the Shh pathway may be related to survival of non-small-cell lung cancer.Univariate analysis revealed that Shh and Gli had significant effects on survival, but multivariate Cox regression analyses failed to identify a factor significantly associated with survival (Table 6).

**Table 6:** **Univariate and multivariate analyses of prognostic factors in non-small-cell lung cancer** ^**a**^

	***P***-value	
Clinicopathological parameter	Univariate analysis ^b^	Multivariate analysis ^c^
Tumor size, <3 cm/>3 cm	0.885	0.108
Visceral pleural invasion, negative/positive	0.357	0.528
Lymph node metastasis, negative/positive	0.495	0.542
Lymphatic thromboembolism, negative/positive	0.976	0.997
Tumor recurrence, negative/positive	0.34	0.959
Distant metastasis, negative/positive	0.591	0.949
Shh expression, negative/positive	0.02^d^	0.959
Gli1 expression, negative/positive	0.055	0.953
LYVE-1 expression, negative/positive	0.323	0.965
VEGFD expression, negative/positive	0.857	0.703

^a^Gli1, Glioma-associated oncogene homolog zinc finger protein 1; LYVE-1, Lymphatic vessel endothelial hyaluronan receptor 1; Shh, Sonic hedgehog; VEGF-D, Vascular endothelial growth factor D. ^b^Logrank test; ^c^Cox regression model; ^d^ *P* < 0.05.

Interestingly, we found LYVE-1 to be associated with lymphatic thromboembolism (Table 5), which is believed to represent the starting point of lymphovascular invasion. However, LYVE-1 was not found to be associated with lymph node invasion or distant metastasis.

## Discussion

The results of the present study show that Shh and Gl-1 are more highly expressed in NSCLC tissues than in normal counterpart tissues.Gli1 protein was detected mainly in tumor tissues, but occasionally in nontumor tissues. These results suggest that the Shh pathway activates NSCLC carcinogenesis, as has been shown for other cancers. The role played by the Shh pathway in lung cancer carcinogenesis has not been fully elucidated, but, in line with other studies on this topic [24, 25], our results demonstrate that the Shh pathway is highly expressed in NSCLC tissues. Raz *et al*. demonstrated that Shh pathway components, such as Shh, Smo and Gli, are strongly related to adenocarcinoma histological cell type in lung cancer, whereas in the present study Gli was strongly expressed in squamous lung cancer. More study of this topic is required.

To our knowledge, this study is the first to show Shh signaling/lymphangiogenic pathway crosstalk in human lung cancer tissues. Furthermore, we found significant relationships between the expression of component of the Shh (Gli1) and lymphangiogenesis (LYVE-1) pathways. Accordingly, the results of this study suggest that the Shh pathway may have a role in lymphangiogenesis.

Asai *et al*. demonstrated that activation of the Shh pathway promotes wound-healing and induces angiogenesis-related events, such as endothelial progenitor cell proliferation, migration, adhesion and tube formation [26]. Bailey *et al*. demonstrated that Shh-neutralizing antibody decreased the number of LYVE-1-positive cells in a mouse model of pancreatic cancer [27]. In our previous study, we demonstrated that the Shh signaling pathway, through the phospho-AKT pathway, enhanced intratumoral lymphatic invasion in gastric cancer [11]. Taken together, these results suggest that the Shh pathway may play a role in the lymphangiogenesis of lung cancer.

We did not find a direct relationship between Shh and lymph node involvement, possibly because of the small number of patients recruited or because Shh affects only the early stage of intratumoral lymphangiogenesis and not lymph node metastasis, which requires the collaborative activities of several systems, such as the epithelial-to-mesenchymal transition and matrix metalloproteinase systems.

Nonetheless, this study shows a strong relationship between LYVE-1 expression and lymphatic thromboembolism, which is considered the first stage of lymph node metastasis. However, the relationship between Shh and lymph node metastasis requires further study.

In the present study, Shh overexpression was found to be associated with the occurrence of visceral pleural invasion. However, the mechanism and underlying significance of this visceral involvement was not determined. Nevertheless, several authors have suggested that activation of the Shh signaling pathway might promote tumor metastasis, and our results are compatible with this suggestion [10, 11, 27].

To our knowledge, we are the first to report that the Shh signaling pathway affects survival in lung cancer. Our results show that expression of the Shh pathway was not affected by TNM stage, tumor recurrence or distant metastasis. Furthermore, despite weak associations between well-known prognostic factors, Shh and Gli expression were found to be associated with survival, which suggests that Shh and Gli expression may have prognostic value independent of TNM staging with respect to the future development of recurrence and distant metastasis. However, we should be cautious about interpreting these results because of the possibility of population selection bias. To avoid this limitation, we selected candidates for surgical resection, but our results should be validated in a larger cohort. To define the mechanism responsible for the survival disadvantage conferred by Shh pathway activation in lung cancer, we examined whether Shh pathway inhibition affects lung cancer cell survival. We found that treatment with siRNA Gli inhibited proliferation of the H1299 and Calu-1 lung cancer cell lines. We tentatively suggest that the poor prognosis of patients with tumors showing high Shh expression is probably due to the induction of proliferation and the activation of lymphangiogenesis.

The significance of the clinicopathological heterogeneity displayed by cancers of the same stage is difficult to understand. Some early-stage lung cancer patients experience recurrence despite complete resection and postoperative adjuvant chemotherapy, whereas others do not. This implies that an understanding of the molecular basis of lung cancer is needed to identify molecular targets for drug development purposes.

In a preclinical study, it was demonstrated that hedgehog inhibitor can have a cytotoxic effect on cancer cells [28]. Recently, it was suggested that the Shh pathway might be involved in drug and radiotherapy resistance in lung cancer [29]. More specifically, it was found that hedgehog pathway inhibitor increases the efficacy of radiotherapy by affecting stromal cells surrounding cancer tissues. The Shh pathway is a key player in embryonal organogenesis and could be a marker for cancer stem cells [30]. Chemo- and radioresistance are characteristic of cancer stem cells. To overcome this resistance, a comprehensive understanding of cancer stem cell markers is needed, because, despite the improved results realized with modern chemotherapeutic and target agents, the outcome of lung cancer remains dismal. Because cure is the final goal of cancer treatment, a new treatment modality that targets cancer stem cells is urgently needed.

## Conclusions

The results of this study show that the Shh signaling pathway affects survival in lung cancer and suggest that Shh initiates lymph node metastasis via LYVE-1-dependent lymphangiogenesis. These findings strongly suggest that the Shh pathway plays an important role in NSCLC progression and that this pathway should be considered a potential therapeutic target. However, somewhat surprisingly, an advanced stage was not found to influence survival, which we attribute to the size of our cohort. Nevertheless, despite the proportion of our cohort that did not have a strong association of survival with well-known prognostic factors such as tumor stage, lymph node stage, tumor recurrence and distant metastasis, the Shh pathway molecule did show a significant difference in survival rate. Further investigations are needed to clarify the role of the Shh signaling pathway and the underlying mechanisms responsible for driving the malignant behavior of NSCLC.

## Abbreviations

Gli: Glioma-associated oncogene homolog zinc finger protein
IHC: Immunohistochemistry
LYVE-1: Lymphatic vessel endothelial hyaluronan receptor 1
MTT: 3-(4,5-dimethylthiazol-2-yl)-2,5-diphenyltetrazolium bromide
NSCLC: Non-small-cell lung cancer
PBS: Phosphate-buffered saline
Ptch: Patched
Shh: Sonic hedgehog
Smo: Smoothened
VEGF-D: Vascular endothelial growth factor D.
